# Interferometric Phase Improvement Based on Polarimetric Data Fusion

**DOI:** 10.3390/s8117172

**Published:** 2008-11-13

**Authors:** Tao Xiong, Jian Yang, Weijie Zhang

**Affiliations:** Department of Electronic Engineering, Tsinghua University, Beijing, 100084, China; E-mails: yangjian_ee@tsinghua.edu.cn (J. Y.); wj-zhang@tsinghua.edu.cn (W. Z.)

**Keywords:** Polarimetric SAR interferometry, phase improvement

## Abstract

In this paper, a method is proposed to improve the interferometric phase quality, based on fusing data from different polarimetric channels. Since lower amplitude implies less reliable phase in general, the phase quality of polarimetric interferometric data can be improved by seeking optimal fusion of data from different polarizations to maximize the resulting amplitude. In the proposed approach, for each pixel, two coherent polarimetric scattering vectors are synchronously projected onto a same optimum direction, maximizing the lower amplitude of the two projections. In the single-look case, the fused phase is equivalent to the weighted average of phases in all polarimetric channels. It provides a good physical explanation of the proposed approach. Without any filtering, the phase noise and the number of residue points are significantly reduced, and the interferometric phase quality is greatly improved. It is a useful tool to preprocess the phase ahead of phase unwrapping. The Cloude's coherence optimization method is used for a comparison. Using the data collected by SIR-C/X-SAR, the authors demonstrate the effectiveness and the robustness of the proposed approach.

## 1. Introduction

Interferometric phase improvement is an important step for Interferometric Synthetic Aperture Radar (InSAR) applications. The original signals collected by a radar system are corrupted by heavy noise, which is caused by the system itself and the propagation. In the traditional SAR interferometry without polarimetric information, several phase filters have been proposed to reduce the noise and improve the phase quality [1-5].

In polarimetric SAR interferometry (PolInSAR), since the scattering element data of each pixel are composed of two scattering matrices or scattering vectors corresponding to two spatially separated antennae, it is possible to enhance the coherence and improve the phase between the signals received by both the antennae. In recent years, several algorithms have been proposed, such as the coherence optimization method with two vectors (CO2) [6, 7], the coherence optimization method with one vector (CO1) [8] and so on. The CO2 method is important for vegetation characteristics analysis. In addition, these methods can be used for phase improvement by interferometric coherence optimization.

In this paper, a novel method is proposed. First we provide a mathematical model to maximize the lower of the two amplitudes from the interferometric complex signal pair. Then the optimal solution is obtained in closed-form. Comparing with the CO2 method, we demonstrate that the proposed method has better performance.

This paper is organized as follows. In Section 2, the coherence optimization method proposed by Cloude *et al.* [6, 7] is reviewed. Section 3 describes the relationship between the amplitude and the phase of a complex signal. In general, weak signals with low amplitudes have unreliable phases. To improve the phase quality, one should augment the amplitude of the signal. For each scattering element, the amplitudes of both the receiving signals should be both as large as possible. The proposed method is introduced in detail in Section 4, where the optimal solution is obtained by an eigendecomposition. In Section 5, a physical explanation is presented. The improved phase is proved to be equivalent to the weighted average of phases in each polarimetric channel in the single-look case, which provides a good intuitive explanation for the proposed approach. Section 6 provides the experimental results, which demonstrate the performance of the proposed method. Finally, some conclusions are given in Section 7.

## 2. Review of coherence optimization (CO2) method

In SAR interferometry, for each scattering element, two complex scalar signals *s*_1_ and *s*_2_ are received from two spatially separated antennae. A 2×2 Hermitian semi-definite coherency matrix [***J***] is defined as:

(1) $$\left[J]=\langle [\begin{array}{c} s_{1}\\ s_{2} \end{array}]\;[s_{1}^{*}s_{1}^{*}]\rangle =[\begin{array}{cc} \langle s_{1}s_{1}^{*}\rangle & \langle s_{1}s_{2}^{*}\rangle \\ \langle s_{2}s_{1}^{*}\rangle & \langle s_{2}s_{2}^{*}\rangle \end{array}\right]$$

where * means the complex conjugation and 〈…〉 indicates the expectation value. From [***J***], the interferometric phase can be obtained by

(2) $$\phi =\arg(s_{1}s_{2}^{*})$$

where arg( ) indicates the argument of a complex number. The interferometric coherence *γ* is defined as

(3) $$\gamma =\frac{\left|\langle s_{1}s_{2}^{*}\rangle \right|}{\sqrt{\langle s_{1}s_{1}^{*}\rangle \langle s_{2}s_{2}^{*}\rangle }},0\leq \gamma \leq 1$$

In polarimetric SAR interferometry, for each scattering element, there are two polarimetric scattering matrices, [***S***_1_] and [***S***_2_], or two scattering vectors

(4) $$k_{i}=\frac{1}{\sqrt{2}}{\left[s_{\text{HH},i}+s_{\text{VV},i},s_{\text{HH},i}-s_{\text{VV},i},s_{\text{HV},i}+s_{\text{VH},i}\right]}^{\text{T}},i=1,2$$

where ^T^ indicates the matrix transposition operation, and *s_pq_* (*p*, *q* = H or V) is the complex scattering coefficient for *q* transmitted and *p* received polarizations in the HV-polarimetric basis. Here, we only consider the reciprocal case, i.e., *s*_HV_ = *s*_VH_.

Similar to [***J***], the 6×6 coherency matrix [***T***[ is defined as [6][7]

(5) $$\left[T]\langle [\begin{array}{c} k_{1}\\ k_{2} \end{array}]\;[k_{1}^{\text{H}}k_{2}^{\text{H}}]\rangle =[\begin{array}{cc} \left[T_{11}\right] & \left[\Omega_{12}\right]\\ \left[\Omega_{21}\right] & \left[T_{22}\right] \end{array}\right]$$

where ^H^ denotes the complex conjugation and transpose.

To extend the scalar formulation into a vector expression, two normalized complex vectors ***w***_1_ and ***w***_2_ are introduced. Then two scattering coefficients *μ*_1_ and *μ*_2_ are defined as the projections of the scattering vectors ***k***_1_ and ***k***_2_ onto the vectors ***w***_1_ and ***w***_2_, respectively,

(6) $$\mu_{1}=w_{1}^{\text{H}}k_{1},\mu_{2}=w_{2}^{\text{H}}k_{2}$$

Then the interferometric phase is derived as

(7) $$\phi_{\text{s}}=\arg(\mu_{1}\mu_{2}^{*})=\arg(w_{1}^{\text{H}}k_{1}k_{2}^{\text{H}}w_{2})$$

for the single-look (SL) case, and

(8) $$\phi_{\text{m}}=\arg(\langle \mu_{1}\mu_{2}^{*}\rangle )=\arg(w_{1}^{\text{H}}[\Omega_{12}]w_{2})$$

for the multi-look (ML) case.

The generalized vector expression for the coherence *γ* is then given by

(9) $$\gamma =\frac{\left|\langle w_{1}^{\text{H}}[\Omega_{12}]w_{2}\rangle \right|}{\sqrt{\langle w_{1}^{\text{H}}[T_{11}]w_{1}\rangle \;\langle w_{2}^{\text{H}}[T_{22}]w_{2}\rangle }}$$

To maximize the coherence *γ*, the Lagrange multiplier method is used to transform the problem into two eigendecompositions [6][7]

(10) $$\begin{array}{l} {\left[T_{11}\right]}^{-1}[\Omega_{12}]{\left[T_{22}\right]}^{-1}{\left[\Omega_{12}\right]}^{\text{H}}w_{1}=vw_{1}\\ {\left[T_{22}\right]}^{-1}{\left[\Omega_{12}\right]}^{\text{H}}{\left[T_{11}\right]}^{-1}[\Omega_{12}]w_{2}=vw_{2} \end{array}$$

The maximum coherence value is then given by the square root of the maximum eigenvalue [6]

(11) $$\gamma_{\max}=\sqrt{v_{\max}}$$

and the corresponding optimum eigenvectors of (10) are ***w***_1opt_ and ***w***_2opt_.

Finally, a sensible constraint is to require

(12) $$\arg(w_{1\text{opt}}^{\text{H}}w_{2\text{pot}})=0$$

In this method, the interferometric coherence *γ* is optimized directly and the maximal coherence value can be obtained by ***w***_1opt_ and ***w***_2opt_. The corresponding interferometric phase *ϕ* defined in (8) is much better than the original phase in each polarimetric channel. The authors derived a decomposition of target scattering characteristics. It is one of the most important methods to explore the scattering structure and behavior of the vegetation-covered area.

Though the coherence might indicate the phase noise, however, it is usually estimated by using neighborhood information and not accurate. So for phase improvement, coherence optimization is not the best approach. Especially in weak signal area, the improved phase by coherence optimization is still noisy. Fortunately, the proposed method can be used to obtain a nearly noise-free phase result in the moderate noise case.

## 3. Relationship between the amplitude and the phase of a complex signal

In SAR interferometry, only one polarimetric channel signal can be received, e.g., HH. For each scattering element, the amplitudes of the complex signals *s*_1_ and *s*_2_ vary with the terrain fluctuation and the scattering characteristic of the ground targets. In some areas, the amplitude of the received signals may be very low. When a complex noise is added to a weak signal, a considerable change in the signal phase may occur. In this case, the interferometric phase between two weak signals will be severely affected by noises and will be of low quality and unreliable. Therefore, a lot of residue points may exist to deteriorate the performance of phase unwrapping. In addition, weak signals usually imply a low signal-to-noise ratio (SNR). The noise components in *s*_1_ and *s*_2_ are totally irrelevant (in repeat-pass interferometry mode). According to (3), the coherence between *s*_1_ and *s*_2_ is corrupted by noise and the interferometric phase between two weak signals (or at least one weak signal) is not reliable, i.e., the quality is low.

The purpose of the proposed method is to fuse the interferometric signal pair in each polarimetric channel to augment the amplitude of the signals, especially in weak signal area. In general, except the effect of decorrelation, most residue points are caused by weak signals. Therefore, optimizing amplitude is necessary and effective to improve the phase quality and eliminate the residue points.

## 4. Amplitude optimization (AO) method

### 4.1. Model

To improve the phase quality and remove the residue points, a feasible way is to augment the amplitudes of both coherent signals.

In polarimetric SAR interferometry, as mentioned above, each scattering element has two polarimetric scattering vectors ***k***_1_ and ***k***_2_. To extend the scalar formulation into a vector expression, as a similar way to the CO2 method, a normalized complex vectors ***w*** is introduced. Then two scattering coefficients *η*_1_ and *η*_2_ are defined as the projections of the scattering vectors ***k*_1_** and ***k*_2_** onto the vector ***w***, respectively

(13) $$\eta_{i}=w^{\text{H}}k_{i},i=1,2$$

The goal of the proposed method is to figure out an optimum vector ***w*** to optimize the amplitude of *η*_1_ and *η*_2_ simultaneously. In other words, the lower amplitude between *η*_1_ and *η*_2_ is maximized. According to Section 3, if both the amplitudes are augmented, the interferometric phase quality can be improved.

Mathematically, the above optimization problem is described as follows:

(14) $$\begin{array}{l} \max_{w}(\min(|w^{\text{H}}k_{1}|,|w^{\text{H}}k_{2}|))\\ \text{subject to}\;\|w\|=1 \end{array}$$

### 4.2. Solution

To obtain the analytic solution of the above problem, it can be transformed into an equivalent problem as follows:

(15) $$\begin{array}{ll} \max(a,b) & \\ \text{subject to} & a=\max_{\text{w}}{\left|w^{\text{H}}k_{1}\right|}^{2},\text{if}{\left|w^{\text{H}}k_{1}\right|}^{2}\leq {\left|w^{\text{H}}k_{2}\right|}^{2}\\ & b=\max_{w}{\left|w^{\text{H}}k_{2}\right|}^{2},\text{if}{\left|w^{\text{H}}k_{1}\right|}^{2}>{\left|w^{\text{H}}k_{2}\right|}^{2}\\ & \left\|w\|=1\right\| \end{array}$$

According to the quadratic programming theory, |***w***^H^***k***_1_|^2^and |***w***^H^***k***_1_|^2^ are two quadrics in three dimensional complex space. Each of them has only one local maximum, which is also the global maximum. The solution has two following cases.

Case I: If the global maximum of |***w***^H^***k***_1_|^2^ is equal to *a* or the global maximum of |***w***^H^***k***_2_|^2^ is equal to *b*, then the optimal ***w*** has the same direction as ***k***_1_ or ***k****_2_* which has the lower amplitude:
  (16) $$w^{\left(1\right)}=\{\begin{array}{l} k_{1}/\|k_{1}\|,\text{if}\|k_{1}\|\leq \|k_{2}\|\\ k_{2}/\|k_{2}\|,\text{if}\|k_{1}\|>\|k_{2}\| \end{array}$$
Case II: If the global maximum of |***w***^H^***k***_1_|^2^ is greater than *a* and the global maximum of |***w***^H^***k***_2_|^2^ is greater than *b*, then the optimal solutions of *a* and *b* must be located on the boundary of |***w***^H^***k***_1_|^2^≤ |***w***^H^***k***_2_|^2^ and |***w***^H^***k***_1_|^2^>|***w***^H^***k***_2_|^2^. Therefore, when ***w*** is the optimum projection direction, both the projections must have the same amplitude:
  (17) $$\left|w^{\text{H}}k_{1}|=|w^{\text{H}}k_{2}\right|$$

In this situation, an eigendecomposition method can be used to obtain the analytic solution. The detailed process is described in Appendix A. The solution is

(18) $$w^{\left(2\right)}=\frac{\alpha v_{1}+v_{2}}{\left\|\alpha v_{1}+v_{2}\right\|},\alpha =\sqrt{-\frac{\lambda_{2}}{\lambda_{1}}}\exp\{-\text{j}\arg(v_{1}^{\text{H}}k_{1}k_{1}^{\text{H}}v_{2})\}$$

where *λ*_1_ > 0, *λ_2_* < 0 are two non-zero eigenvalues of matrix ***k***_1_***k***_1_^H^ − ***k***_2_***k***_2_^H^, with corresponding eigenvectors ***v***_1_ and ***v***_2_, respectively.

Appendix A also gives the judgment condition of the two situations. The final solution of the model (14) is

(19) $$w=\{\begin{array}{l} w^{\left(2\right)},\text{if}(\sqrt{\frac{-\lambda_{2}}{\lambda_{1}}}>\max(\frac{\left|v_{1}^{\text{H}}k_{1}\right|}{\left|v_{2}^{\text{H}}k_{1}\right|},\frac{\left|v_{1}^{\text{H}}k_{2}\right|}{\left|v_{2}^{\text{H}}k_{2}\right|}))\text{or}(\sqrt{\frac{-\lambda_{2}}{\lambda_{1}}}<\min(\frac{\left|v_{1}^{\text{H}}k_{1}\right|}{\left|v_{2}^{\text{H}}k_{1}\right|},\frac{\left|v_{1}^{\text{H}}k_{2}\right|}{\left|v_{2}^{\text{H}}k_{2}\right|}))\\ w^{\left(1\right)},\text{else} \end{array}$$

In the single-look case, the fused phase is

(20) $$\phi_{\text{s}}=\arg(\eta_{1}\eta_{2}^{*})=\arg(w^{\text{H}}k_{1}k_{2}^{\text{H}}w)$$

and in the multi-look case, the fused phase is

(21) $$\phi_{\text{m}}=\text{arg}(\langle \eta_{1}\eta_{2}^{*}\rangle )=\arg(w^{\text{H}}[\Omega_{12}]w)$$

## 5. Physical explanation

The cross-correlation item $k_{2}^{\text{H}}k_{1}$ is important, because it contains both the polarimetric and interferometric information. Let *φ* denote the phase of $k_{2}^{\text{H}}k_{1}$, then *φ* can be proved to be equivalent to the fused phase in the single-look case, i.e., *ϕ*_s_ in (20). (See Appendix B.)

(22) $$\phi_{\text{s}}=\arg(k_{2}^{\text{H}}k_{1})=\varphi$$

According to the definition of the scattering vector in (4), the inner product $k_{2}^{\text{H}}k_{1}$ can be expanded as

(23) $$k_{2}^{\text{H}}k_{1}=s_{\text{HH},2}^{*}s_{\text{HH},1}+s_{\text{VV},2}^{*}s_{\text{VV},1}+2s_{\text{HV},2}^{*}s_{\text{HV},1}=|s_{\text{HH},2}^{*}s_{\text{HH},1}|{\text{e}}^{\text{j}\varphi_{\text{HH}}}+|s_{\text{VV},2}^{*}s_{\text{VV},1}|{\text{e}}^{\text{j}\varphi_{\text{VV}}}+2|s_{\text{HV},2}^{*}s_{\text{HV},1}|{\text{e}}^{\text{j}\varphi_{\text{HV}}}$$

where *φ_pq_* denotes the interferometric phase of *pq* channel. It is a weighted average of information in each polarimetric channel. Since |*s*_*pq*,1_| and |*s*_*pq*,2_| denote the amplitudes of the signals, the larger the product of them, the larger the weight. This is reasonable from the basic viewpoint in Section 3: the phase of strong signals is more reliable than that of weak signals in general. Since a larger weight is assigned to a more reliable phase of a given polarimetric channel, the noise of the improved phase is reduced effectively and the coherence is enhanced.

## 6. Experiments and results

### 6.1. Experimental data

Here we used the single-look L-band experimental data consisting of fully polarimetric complex image pairs of the Tien Shan test site acquired by the SIR-C/X-SAR radar system on Oct. 8 and 9, 1994. The test area is close to the southern edge of Lake Baikal, Russia. It contains many different ground targets such as forest, cropland, bare ground and mountain. Without denoising, the interferometric phase is corrupted by heavy noise and lots of residue points exist.

### 6.2. Amplitude vs. phase relationship

Though the coherence parameter has no direct link to the phase, it is usually regarded as a quality descriptor of phase information. Though other parameters such as the phase derivative variance and the maximum phase gradient can also be used to measure phase quality [10], the coherence is more widely accepted in SAR interferometry. In (3), the coherence seems to be independent of the amplitude. However, since lower amplitudes always correspond to lower SNR, uncorrelated noises will dominate the value of the coherence. Therefore, weak signals lead to low coherence.

**Figure 1.**
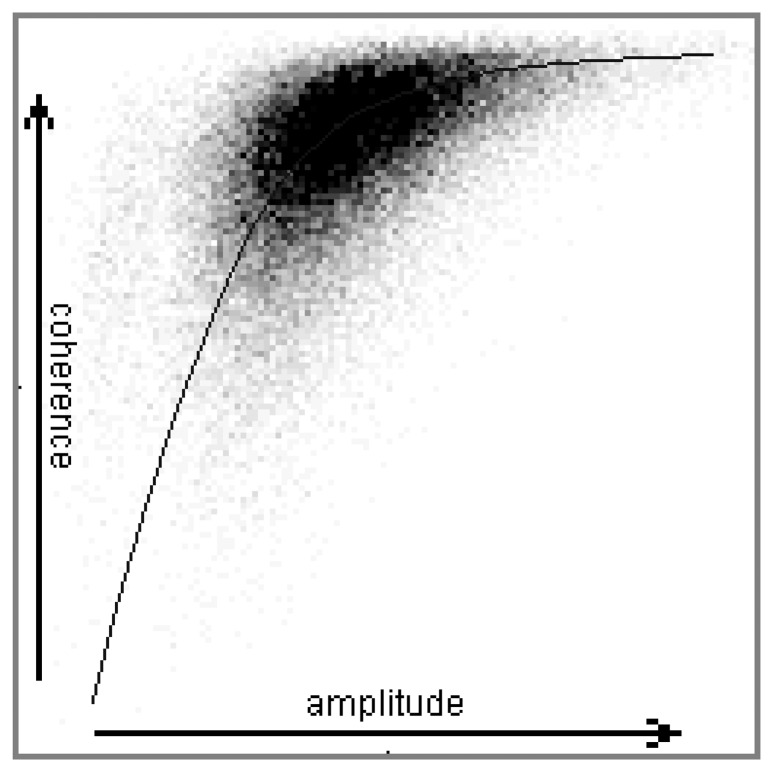
The relationship between the coherence and amplitude. It is a 2-D histogram of the coherence and amplitude in a forest area (10,000 samples).

Now we use the coherence-amplitude map to demonstrate the relationship between the amplitude and the phase of complex signals. 10,000 samples with same scattering characteristics are used to draw the 2-dimensional histogram between coherence and amplitude. Figure 1 shows the forest case as an example. Both the coherence and amplitude have 128 gray levels.

From the distribution we conclude that in most cases, the coherence of weak signals is low, and large amplitudes in general correspond to large coherence. Therefore, a larger amplitude implies a more reliable phase. Another experiment in [9] also verifies this relationship.

### 6.3. Vegetation and bare ground

The scattering mechanism of the vegetation is very complicated due to its multiple components such as leaves, branches, trunks and the underlying ground. According to the vegetation scattering model based on physical properties, the total response of the vegetation is composed of the volume scattering (random or oriented) and the ground scattering (with or without the trunk) [11][12]. Moreover, due to the repeat-pass interferometry mode, the temporal decorrelation can not be neglected, especially in the vegetation-covered area. Therefore, improving the phase quality is necessary for topography retrieval.

**Figure 2.**
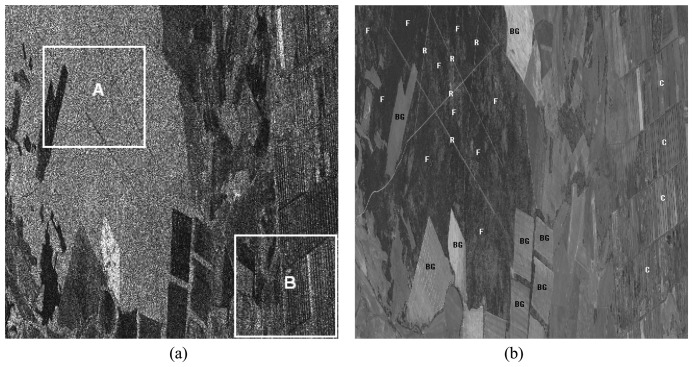
**(a)** The amplitude image in HH channel of the vegetation and bare ground test area. The typical targets in Frame A and B are forest and bare ground, cropland, respectively. **(b)** The corresponding optical image from Google Earth. F, R, GB and C indicate forest, road, bare ground and cropland, respectively.

Figure 2(a) shows the *s*_HH_ of the test area (1,000×1,000 pixels), which includes several different kinds of targets, such as forest (F), road (R), bare ground (BG) and cropland (C). The corresponding optical image from Google Earth with the same resolution is given as Figure 2(b).

To demonstrate the effectiveness of the proposed method, two areas in white frame A and frame B containing typical targets are enlarged and processed.

**Figure 3.**
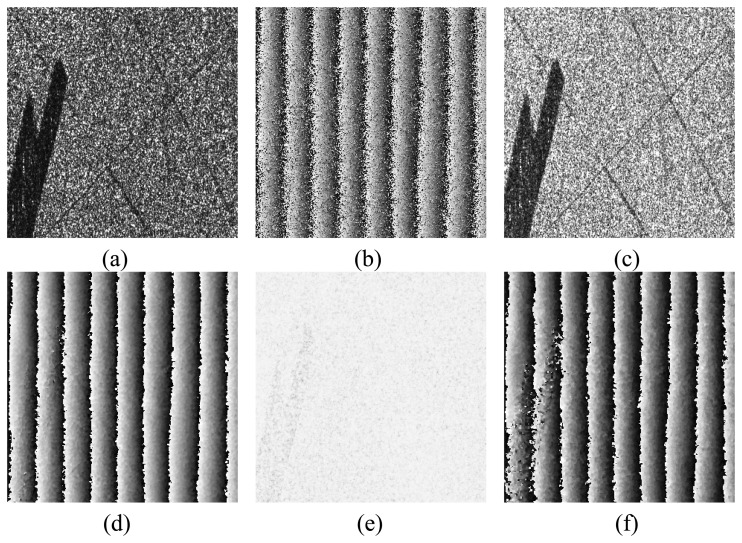
**(a)** The amplitude in HH channel of the enlarged area from Frame A in Figure 2(a). **(b)** The phase in HH channel. **(c)** The amplitude obtained by the AO method in the ML case. **(d)** The phase obtained by the AO method in the ML case. **(e)** The coherence obtained by the CO2 method in the ML case. **(f)** The phase obtained by the CO2 method in the ML case.

The enlarged version of frame A is shown in Figure 3(a). The ground is mostly covered by forest with three roads through it. The black area is the bare ground. With noise and the effect of decorrelation, the phase noise in HH channel is so heavy that all details of the terrain are submerged (Figure 3(b)). The averages of lower amplitudes of the selected area in HH, HV and VV channels are 0.3604, 0.1401, and 0.2578, respectively. Using the proposed method, the average of lower amplitude of the fused image pair is improved to 0.4768 and the amplitude of *η*_1_ in (13) is shown in Figure 3(c). It is obviously “whiter” than the amplitude of HH channel. The fused phase in (21), using 3 × 3 window, is shown in Figure 3(d). Noise is removed obviously and the phase fringes can be clearly observed. 99.76% residue points are removed. The improved phase between the forest area and the bare ground has no obviously boundary. It implies that the phases in the forest area can be regarded as those of the underlying topography, since the phase in the bare ground definitely corresponds to the topography.

To make a comparison, the phase result by the coherence optimization (CO2) method is also calculated and shown in Figure 3(f). The coherence is optimized close to 1 (shown in Figure 3(e), the white and the black colors mean 1 and 0, respectively) and many noises in the forested area are removed. However, the phase of the bare ground is still noisy, which is not in accordance with the “flat property” of the bare ground as shown in the optical image.

Table 1 lists more comparisons between the amplitude optimization (AO) method and the coherence optimization (CO2) method. It includes the average of lower amplitude, the mean coherence, and the residue point number in both the single-look (SL) and multi-look (ML) cases.

**Figure 4.**
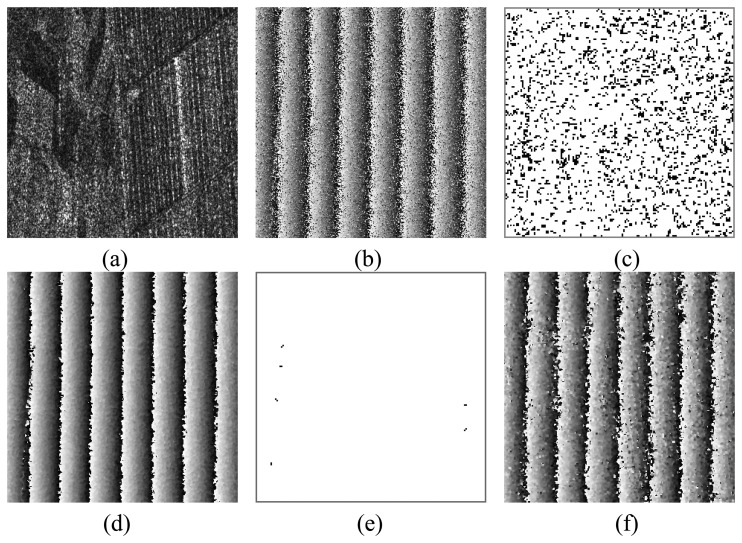
**(a)** The amplitude in HH channel of the enlarged area from Frame B in Figure 2(a). **(b)** The phase in HH channel. **(c)** The residue map in HH channel. **(d)** The phase obtained by the AO method in the ML case. **(e)** The residue map of the phase obtained by the AO method in the ML case. **(f)** The phase obtained by the CO2 method in the ML case.

Figure 4(a) shows the enlarged area of Frame B. Most parts of the ground are covered by lowvegetation like the crop and grass. The parallel straight lines are possible ridges of field. The dihedral angle between the ridge and the ground leads to strong responded signals. The phase in HH channel [Figure 4(b)] is so noisy that it yields 6,550 residue points as shown in Figure 4(c) (black points). The improved phase by the proposed AO method and the CO2 method are given in Figure 4(d) and (f), respectively. Obviously, the phase improved by the AO method has the better quality with less noise. 99.63% residue points are removed successfully as shown in Figure 4(e), demonstrating the effectiveness of the proposed approach.

### 6.4. Mountain

Phase unwrapping (PU) is the key step of digital elevation model (DEM) generation. The main difficulties of phase unwrapping are from noise and steep topography. Both the factors lead to the existence of huge amount of residue points. For path following based PU methods, branch cuts are used to balance the charge of the positive and negative residue points. In the case that a large numbers of dense residue points exist, several branch cuts based algorithms [13, 14] do not work.

Though Buckland [15] proposed an algorithm based on the Hungarian algorithm from integer programming and declared the algorithm enables one to unwrap unfiltered speckle-interferometry phase maps at high point densities (0.1 points per pixel), the computation efficiency should be considered. It will take a long time to solve a large wrapped phase map with heavy noise. So it is significant to improve the phase quality before phase unwrapping.

**Figure 5.**
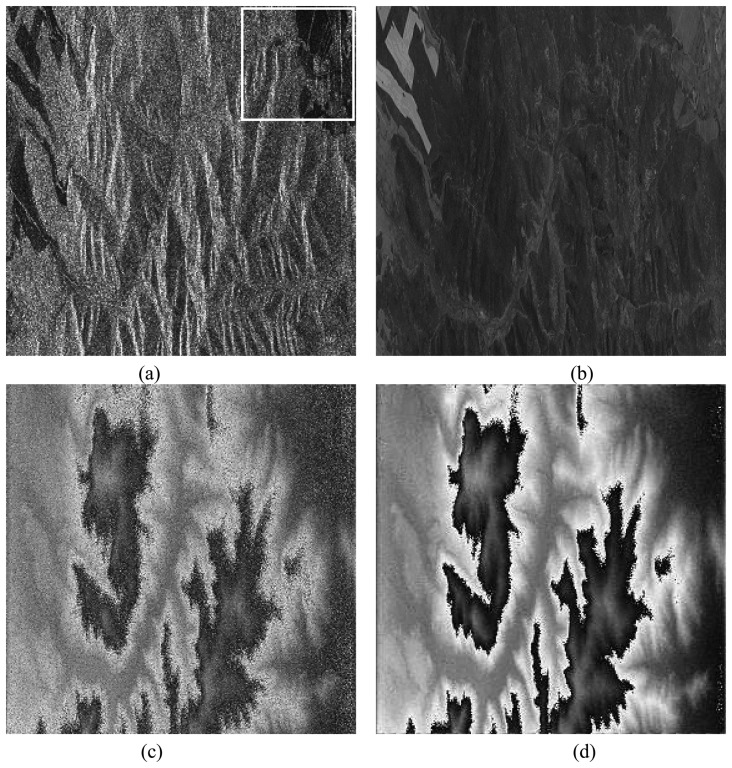
**(a)** The amplitude image in HH channel of mountain test area. **(b)** The corresponding optical image from Google Earth. **(c)** The phase in HH channel. **(d)** The phase obtained by the AO method in the ML case.

Figure 5(a) shows a test area containing a mountainous area. The corresponding optical image is shown in Figure 5(b), which is also from Google Earth. The topography is not steep. The interferometric phase in HH channel is displayed in Figure 5(c). With 1,000×1,000 pixels, the phase map after flat-removal corresponds to 95,329 residue points, so the density of residue points is close to 0.1 sources per pixel. Though a reasonable result may be figured out by the algorithm in [15], it is time-costly and the unwrapped phase is still noisy.

**Figure 6.**
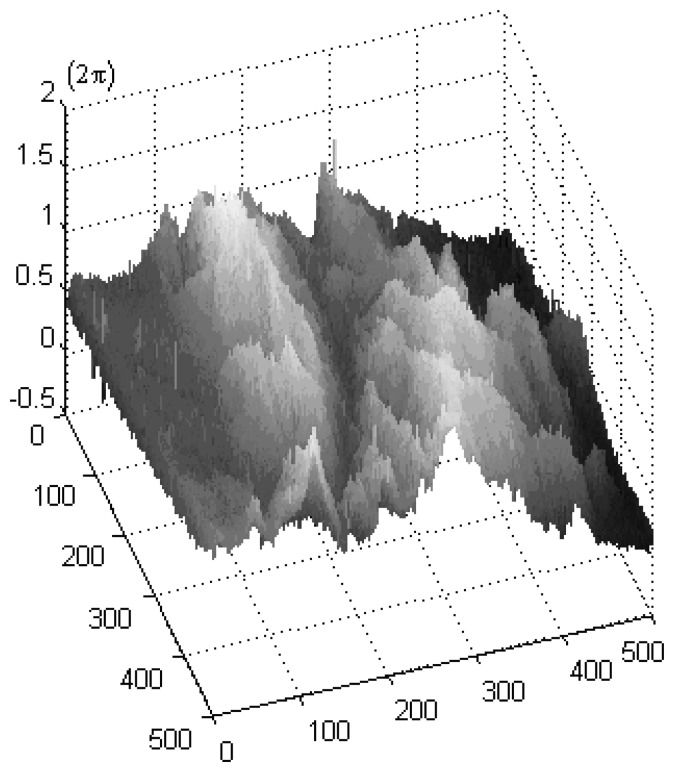
The unwrapped phase 3-D illustration corresponding to Figure 5(d).

Fusing the information from each polarimetric channel, the average of lower amplitude of the image pair is enhanced from 0.3250 (in HH), 0.1310 (in HV) and 0.2831 (in VV) to 0.4574. The fused phase is shown in Figure 5(d). 99.96% residue points are removed. Using typical PU algorithms, the unwrapped phase can be obtained fast and accurately. The 3-D illustration is displayed in Figure 6. From Figure 5(d), the topography becomes clearer and the detailed information is preserved well.

**Figure 7.**
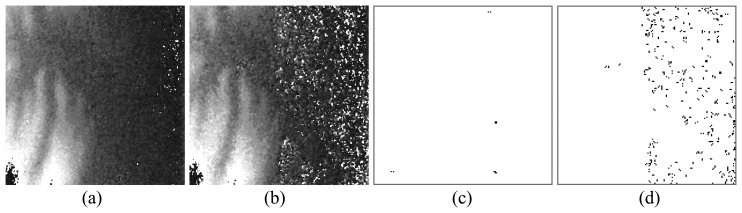
**(a)** The phase obtained by the AO method, which corresponds to the area from the frame in Figure 5(a). **(b)** The phase obtained by the CO2 method. **(c)** The residue map of the phase obtained by the AO method. **(d)** The residue map of the phase obtained by the CO2 method. All of them are in the ML case.

The CO2 method can be used to enhance the phase quality well in most mountainous areas with moderate and strong signals. But in flat ground area with weak signals, the fused phases still correspond to lots of residue points. Please pay attention to the area in the white frame in Figure 5(a) that the amplitudes of the right half pixels are low. The improved phases obtained by the proposed AO method and the CO2 method are shown in Figure 7(a) and (b), respectively. Corresponding to the area with low amplitude, lots of residue points exist in the right half of Figure 7(d) and the phase in Figure 7(b) is noisy.

Based on the signal amplitude optimization, the phase result corresponds to very few residue points in both strong signal areas and weak signal areas shown in Figure 7(c). It demonstrates the robustness of the proposed method.

To demonstrate the denoising ability of the AO method further, we add some noise to the original PolInSAR data in the white frame in Figure 5(a) for simulation. In each pixel, let $s_{i}={\left[s_{\text{HH},i},{\sqrt{2s}}_{\text{HV},i},s_{\text{VV},i}\right]}^{\text{T}}$, *i* = 1, 2, then the covariance matrix by each antenna, $[C_{i}]=\langle s_{i}s_{i}^{\text{H}}\rangle$, is calculated. According to the noise statistics in [16], the simulated complex noise vector ***n****_i_* has complex Gaussian distribution *N* (0, *m*[***C****_i_*]), where *m* is a scalar between 0 and 1. The residue point number (before flat-removal) is used to measure the performance of both the methods. *m* can be regarded as an indicator of the added noise intensity.

**Figure 8.**
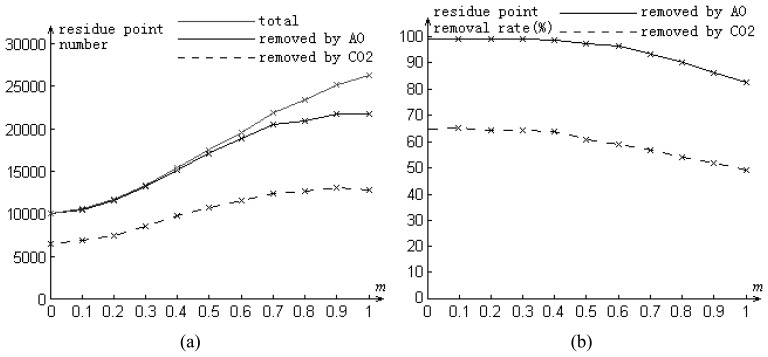
**(a)** The comparison of the removed residue point numbers obtained by the AO and CO2 methods at different simulated noise level *m* (in the ML case). The test area is from the frame in Figure 5(a). **(b)** The corresponding residue point removal rates.

**Figure 9.**
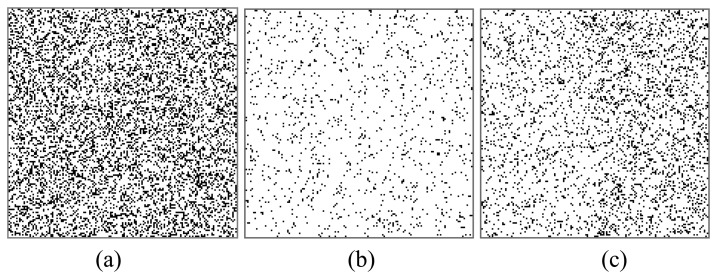
**(a)** The residue point map in HH channel with strong noise (*m* = 1). **(b)** The residue point map of the phase obtained by the AO method in the ML case. **(c)** The residue point map of the phase obtained by the CO2 method in the ML case.

Figure 8 illustrates the denoising performance of the AO and CO2 methods when *m* increases from 0 to 1. When the noise is comparatively weak, e.g., *m* ≤ 0.3, more than 99% residue points are removed by the AO method. Even in the strong noise case, e.g., *m*≥0.8 (shown in Figure 9(a)), the noise can be reduced effectively and the spatial distribution of the remained residue points is close to uniform (Figure 9(b)), regardless of strong signal area (mountain) or weak area (bare ground). On the other hand, using the CO2 method, the remained residue points concentrate in bare ground area (Figure 9(c)). It may be difficult to unwrap the phase with such dense residue points. For example, in the case of *m* = 1, the average density of residue points is 0.135 points per pixel by the CO2 method (Figure 9(c)). Most existing PU methods do not work in such an extreme situation. Using the AO method, the average density can be reduced to 0.049 points per pixel (Figure 9(b)). Then the unwrapped phase can be obtained by several noise-immune methods.

## 7. Conclusions

A novel interferometric phase improvement method has been proposed. The key point is to maximize the amplitude of the signals based on the relationship between the amplitude and the phase of a complex signal. In the single look case, the improved phase is equivalent to the average of information in each polarimetric channel with different weights which are proportional to the amplitude in each channel.

In the proposed method, we used one normalized complex vector instead of two, because the correlation information between both the interferometric channels is important, and the proposed method did not optimize the coherence directly. In one-vector case, the correlation information is used more sufficiently, contained in the eigenvectors of matrix ***k***_1_***k***_1_^H^ − ***k***_2_***k***_2_^H^. Considering two-vector case, ***w***_1_ and ***w***_2_ can be figured out as the normalized version of ***k***_1_ and ***k***_2_, respectively, with the only constraint (12). More correlation information leads to better result.

Using the PolInSAR data, the performance of phase improvement has been demonstrated. In the multi-look case, more than 99% residue points caused by moderate noise can be removed by the proposed method in both strong and weak signal areas. The detailed information of topography is observed more clearly, which makes phase unwrapping becomes easier and faster.

## Figures and Tables

**Table 1.** The comparisons of average lower amplitude, average coherence and residue point number among the original HH/HV/VV channel data and the fused data processed by the AO and CO2 method in both the single-look and multi-look cases in Figure 3(a).

	Lower Amplitude	Coherence	Residue point number

**HH**	0.3604	0.7843	8331
**HV**	0.1401	0.7077	12033
**VV**	0.2578	0.7450	10279
**AO in SL**	0.4768	0.8523	1699
**CO2 in SL**	0.2795	0.8858	1889
**AO in ML**	---------	0.8228	20
**CO2 in ML**	---------	0.9634	173

**Figure A1.**
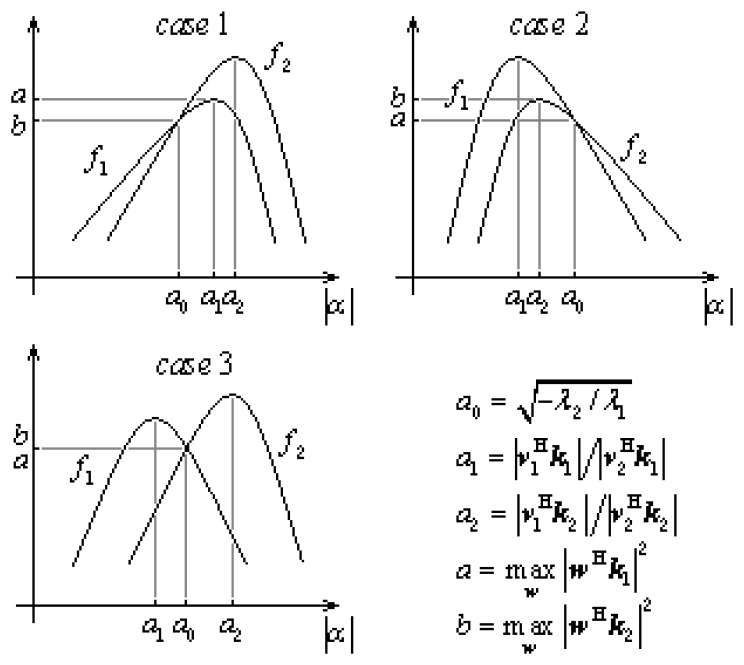
The sketch map of the three sub-cases of the model solution in the second case.

## Appendix A

### Solution of the Amplitude Optimization Model

In Case II, from (17), it can be derived that

(A1) $$w^{\text{H}}(k_{1}k_{1}^{\text{H}}-k_{2}k_{2}^{\text{H}})w=0$$

If ***k***_1_ = *c****k****_2_* (*c* is an arbitrary complex number except zero), the case can be categorized into Case I.

If ***k***_1_ ≠ *c****k****_2_*, let [***A***] denote matrix $k_{1}k_{1}^{\text{H}}-k_{2}k_{2}^{\text{H}})$, then the rank of [***A***] is equal to 2. Moreover, [***A***] is an indefinite matrix. So it has three eigenvalues *λ*_1_ > 0, *λ*_2_ < 0 and *λ*_3_ = 0, and the corresponding eigenvectors are ***v***_1_, ***v***_2_ and ***v***_3_, respectively.

If ***w***= ***v***_3_, it completely meets (A1) due to *λ*_3_ = 0. But the space spanned by ***k***_1_ and ***k***_2_, i.e., *Span*{***k***_1_,***k***_2_}, is equivalent to *Span*{***v***_1_, ***v***_2_}. Since ***v***_3_ is orthogonalized to both ***v***_1_ and ***v***_2_, then |***w***^H^***k***_1_| = |***w***^H^***k***_2_| = 0. It does not satisfy the goal of (14).

Thus, ***w*** can be expressed as the linear combination of ***v***_1_ and ***v****_2_*

(A2) $$w=\beta (\alpha v_{1}+v_{2})$$

where *α* is a complex coefficient and *β* is a real normalized coefficient. Substituting (A2) into (A1),

(A3) $$\begin{array}{l} w^{\text{H}}(k_{1}k_{1}^{\text{H}}-k_{2}k_{2}^{\text{H}})w=\beta^{2}{\left(\alpha v_{1}+v_{2}\right)}^{\text{H}}(\lambda_{1}v_{1}v_{1}^{\text{H}}+\lambda_{2}v_{2}v_{2}^{\text{H}})(\alpha v_{1}+v_{2})\\ =\beta^{2}({\left|\alpha \right|}^{2}v_{1}^{\text{H}}v_{1}v_{1}^{\text{H}}v_{1}\lambda_{1}+v_{2}^{\text{H}}v_{2}v_{2}^{\text{H}}v_{2}\lambda_{2})=\beta^{2}({\left|\alpha \right|}^{2}{\left\|v_{1}\right\|}_{2}^{4}\lambda_{1}+{\left\|v_{2}\right\|}_{2}^{4}\lambda_{2})=0 \end{array}$$

So

(A4) $$|\alpha |=\sqrt{-\frac{\lambda_{2}}{\lambda_{1}}}\frac{{\left\|v_{2}\right\|}_{2}^{2}}{{\left\|v_{1}\right\|}_{1}^{2}}=\sqrt{-\frac{\lambda_{2}}{\lambda_{1}}}$$

Substituting (A2) into $w^{\text{H}}k_{1}k_{1}^{\text{H}}w$,

(A5) $$\begin{array}{l} w^{\text{H}}k_{1}k_{1}^{\text{H}}w=\beta^{2}{\left(\alpha v_{1}+v_{2}\right)}^{\text{H}}k_{1}k_{1}^{\text{H}}(\alpha v_{1}+v_{2})\\ =\beta^{2}({\left|\alpha \right|}^{2}{\left|v_{1}^{\text{H}}k_{1}\right|}^{2}+\alpha v_{2}^{\text{H}}k_{1}k_{1}^{\text{H}}v_{1}+{\left(\alpha v_{2}^{\text{H}}k_{1}k_{1}^{\text{H}}v_{1}\right)}^{*}+{\left|v_{2}^{\text{H}}k_{1}\right|}^{2}) \end{array}$$

According to the Cauchy inequality, the maximum of (A5) corresponds to $\alpha =dv_{1}^{\text{H}}k_{1}k_{1}^{\text{H}}v_{2}$, where *d* is an nonnegative real number. So the argument of α is

(A6) $$\arg(\alpha )=\arg(v_{1}^{\text{H}}k_{1}k_{1}^{\text{H}}v_{2})$$

After substituting (A4) and (A6) into (A2) and normalization, one finally obtains:

(A7) $$w^{\left(2\right)}=\frac{\alpha v_{1}+v_{2}}{\left\|\alpha v_{1}+v_{2}\right\|},\alpha =\sqrt{-\frac{\lambda_{2}}{\lambda_{1}}}\exp\{-\text{j}\arg(v_{1}^{\text{H}}k_{1}k_{1}^{\text{H}}v_{2})\}$$

To determine how to choose ***w***^(1)^ and ***w***^(2)^, we define two functions *f*_1_(|*α*|) and *f*_2_(|*α*|) with only one variable |*α*|

(A8) $$\begin{array}{l} f_{i}(|\alpha |)=\frac{{\left|w^{\text{H}}k_{i}\right|}^{2}}{{\left\|w\right\|}^{2}}=\frac{{\left|{\left(\alpha v_{1}+v_{2}\right)}^{\text{H}}k_{i}\right|}^{2}}{{\left\|\alpha v_{1}+v_{2}\right\|}^{2}}=\frac{{\left|\alpha \right|}^{2}{\left|v_{1}^{\text{H}}k_{i}\right|}^{2}+\alpha^{*}v_{1}^{\text{H}}k_{i}k_{1}^{\text{H}}v_{2}+\alpha v_{2}^{\text{H}}k_{i}k_{i}^{\text{H}}v_{1}+{\left|v_{2}^{\text{H}}k_{i}\right|}^{2}}{{\left|\alpha \right|}^{2}{\left\|v_{1}\right\|}^{2}+\alpha^{*}v_{1}^{\text{H}}v_{2}+\alpha v_{2}^{\text{H}}v_{1}+{\left\|v_{2}\right\|}^{2}}\\ =\frac{{\left(|\alpha ||v_{1}^{\text{H}}k_{i}|+|v_{2}^{\text{H}}k_{i}|\right)}^{2}}{{\left|\alpha \right|}^{2}{\left\|v_{1}\right\|}^{2}+{\left\|v_{2}\right\|}^{2}},i=1,2 \end{array}$$

Let

(A9) $$\begin{array}{l} \frac{\text{d}f_{i}(|\alpha |)}{\text{d}|\alpha |}=\text{d}\frac{{\left(|\alpha ||v_{1}^{\text{H}}k_{i}|+|v_{2}^{\text{H}}k_{i}|\right)}^{2}}{{\left|\alpha \right|}^{2}{\left\|v_{1}\right\|}^{2}+{\left\|v_{2}\right\|}^{2}}/\text{d}|\alpha |=\text{d}\frac{z{\left|\alpha \right|}^{2}+w|\alpha |+u}{x{\left|\alpha \right|}^{2}+y}/\text{d}|\alpha |\\ =\frac{\left(2z|\alpha |+w)(x{\left|\alpha \right|}^{2}+y)-2|\alpha |x(z{\left|\alpha \right|}^{2}+w|\alpha |+u\right)}{{\left(x{\left|\alpha \right|}^{2}+y\right)}^{2}}=0 \end{array}$$

then

(A10) $$wx{\left|\alpha \right|}^{2}+2(xu-zy)|\alpha |-wy=0$$

The corresponding solution of |*α*| is

(A11) $$|\alpha |\frac{2zy}{wx}=\frac{\left|v_{1}^{\text{H}}k_{i}\right|}{\left|v_{2}^{\text{H}}k_{i}\right|}>0$$

Because of the monotonicities of *f*_1_ and *f*_2_, there are three sub-cases as follows (schematically shown in Figure A1):

1) If $\left|\alpha |<|v_{1}^{\text{H}}k_{1}|/|v_{2}^{\text{H}}k_{1}|\text{and}|\alpha |<|v_{1}^{\text{H}}k_{2}|/|v_{2}^{\text{H}}k_{2}\right|$, *a* is greater than *b* and ***w***= ***w***^(1)^;
2) If $\left|\alpha |>|v_{1}^{\text{H}}k_{1}|/|v_{2}^{\text{H}}k_{1}|\text{and}|\alpha |>|v_{1}^{\text{H}}k_{2}|/|v_{2}^{\text{H}}k_{2}\right|$, *b* is greater than *a* and ***w***= ***w***^(1)^;
3) Otherwise, ***w***= ***w***^(1)^,

where *a* and *b* are defined in (15).

In conclusion, the final solution of the model is

(A12) $$w=\{\begin{array}{l} w^{\left(2\right)},\text{if}(\sqrt{\frac{-\lambda_{2}}{\lambda_{1}}}>\max(\frac{\left|v_{1}^{\text{H}}k_{1}\right|}{\left|v_{2}^{\text{H}}k_{1}\right|},\frac{\left|v_{1}^{\text{H}}k_{2}\right|}{\left|v_{2}^{\text{H}}k_{2}\right|}))\text{or}(\sqrt{\frac{-\lambda_{2}}{\lambda_{1}}}<\min(\frac{\left|v_{1}^{\text{H}}k_{1}\right|}{\left|v_{2}^{\text{H}}k_{1}\right|},\frac{\left|v_{1}^{\text{H}}k_{2}\right|}{\left|v_{2}^{\text{H}}k_{2}\right|}))\\ w^{\left(1\right)},\text{else} \end{array}$$

## Appendix B

### Proof of Equation (22)

According to (22), can be rewritten as

(B1) $$\varphi =\arg(k_{2}^{\text{H}}k_{1})$$

From (19) and (20), if ***w*** = ***w***^(1)^, then

(B2) $$\phi_{\text{s}}=\arg(mk_{2}^{\text{H}}k_{1})$$

where *m* is equal to ║***k***_1_║^2^ or ║***k***_2_║^2^. So *ϕ*_s_ and *φ* are equivalent. If ***w*** = ***w***^(2)^, since ***v***_1_ is an eigenvector of the matrix $k_{1}k_{1}^{\text{H}}-k_{2}k_{2}^{\text{H}}$, i.e., $(k_{1}k_{1}^{\text{H}}-k_{2}k_{2}^{\text{H}})v_{1}=\lambda_{1}v_{1}$ then

(B3) $$v_{1}^{\text{H}}k_{1}k_{2}^{\text{H}}(k_{1}k_{1}^{\text{H}}-k_{2}k_{2}^{\text{H}})v_{1}=\lambda_{1}v_{1}^{\text{H}}k_{1}k_{2}^{\text{H}}v_{1}\Rightarrow v_{1}^{\text{H}}k_{1}k_{2}^{\text{H}}v_{1}=\frac{{\left|k_{1}^{\text{H}}v_{1}\right|}^{2}}{\lambda_{1}+{\left\|k_{2}\right\|}_{2}^{2}}k_{2}^{\text{H}}k_{1}$$

and

(B4) $$v_{2}^{\text{H}}k_{1}k_{2}^{\text{H}}(k_{1}k_{1}^{\text{H}}-k_{2}k_{2}^{\text{H}})v_{1}=\lambda_{1}v_{2}^{\text{H}}k_{1}k_{2}^{\text{H}}v_{1}\Rightarrow v_{2}^{\text{H}}k_{1}k_{2}^{\text{H}}v_{1}=\frac{v_{2}^{\text{H}}k_{1}k_{1}^{\text{H}}v_{1}}{\lambda_{1}{\left\|k_{2}\right\|}_{2}^{2}}k_{2}^{\text{H}}k_{1}$$

Similarly,

(B5) $$v_{2}^{\text{H}}k_{1}k_{2}^{\text{H}}v_{2}=\frac{{\left|k_{1}^{\text{H}}v_{2}\right|}^{2}}{\lambda_{2}+{\left\|k_{2}\right\|}_{2}^{2}}k_{2}^{\text{H}}k_{1}$$

and

(B6) $$v_{1}^{\text{H}}k_{1}k_{2}^{\text{H}}v_{2}=\frac{v_{1}^{\text{H}}k_{1}k_{1}^{\text{H}}v_{2}}{\lambda_{2}+{\left\|k_{2}\right\|}_{2}^{2}}k_{2}^{\text{H}}k_{1}$$

According to (A5), $w^{\text{H}}k_{1}k_{2}^{\text{H}}w$ can be written as

(B7) $$w^{\text{H}}k_{1}k_{2}^{\text{H}}w=\frac{{\left|\alpha \right|}^{2}v_{1}^{\text{H}}k_{1}k_{2}^{\text{H}}v_{1}+\alpha^{*}v_{1}^{\text{H}}k_{1}k_{2}^{\text{H}}v_{2}+\alpha v_{2}^{\text{H}}k_{1}k_{2}^{\text{H}}v_{1}+v_{2}^{\text{H}}k_{1}k_{2}^{\text{H}}v_{2}}{{\left\|\alpha v_{1}+v_{2}\right\|}^{2}}$$

Substituting (B3) ∼ (B6) into (B7), we can simplify the formulation and obtain:

(B8) $$w^{\text{H}}k_{1}k_{2}^{\text{H}}w=(\frac{{\left|\alpha \right|}^{2}{\left|k_{1}^{\text{H}}v_{1}\right|}^{2}}{\lambda_{1}+{\left\|k_{2}\right\|}_{2}^{2}}+\frac{\left|\alpha ||v_{1}^{\text{H}}k_{1}k_{1}^{\text{H}}v_{2}\right|}{\lambda_{2}+{\left\|k_{2}\right\|}_{2}^{2}}+\frac{\left|\alpha ||v_{2}^{\text{H}}k_{1}k_{1}^{\text{H}}v_{1}\right|}{\lambda_{1}+{\left\|k_{2}\right\|}_{2}^{2}}+\frac{{\left|k_{1}^{\text{H}}v_{2}\right|}^{2}}{\lambda_{2}+{\left\|k_{2}\right\|}_{2}^{2}})\frac{k_{2}^{\text{H}}k_{1}}{{\left\|\alpha v_{1}+v_{2}\right\|}^{2}}=lk_{2}^{\text{H}}k_{1}$$

where *l* is a positive real number. So (20) can be rewritten as

(B9) $$\phi_{\text{s}}=\arg(lk_{2}^{\text{H}}k_{1})$$

then *ϕ*_s_ and φ are equivalent.
